# Health-Related Quality of Life during Chemoradiation in Locally Advanced Rectal Cancer: Impacts and Ethnic Disparities

**DOI:** 10.3390/cancers11091263

**Published:** 2019-08-28

**Authors:** Stephanie Hui-Su Lim, Emilia Ip, Weng Ng, Wei Chua, Ray Asghari, Aflah Roohullah, Joseph Descallar, Christopher Henderson, Kevin Spring, Paul de Souza, Madeleine T. King

**Affiliations:** 1 Medical Oncology Group, Ingham Institute for Applied Medical Research, Liverpool, New South Wales 2170, Australia; 2 Department of Medical Oncology, Macarthur Cancer Therapy Centre, Campbelltown, New South Wales 2560, Australia; 3 University of New South Wales, Kensington, New South Wales 2052, Australia; 4 Western Sydney University, Liverpool, New South Wales 2170, Australia; 5 Department of Medical Oncology, Liverpool Hospital, Liverpool, New South Wales 2170, Australia; 6 Department of Medical Oncology, Bankstown Hospital, Bankstown, New South Wales 2200, Australia; 7 Department of Anatomical Pathology, Liverpool Hospital, Liverpool, New South Wales 2170, Australia; 8 School of Psychology, University of Sydney, Camperdown, New South Wales 2006, Australia

**Keywords:** health-related quality of life, chemoradiation, rectal cancer

## Abstract

*Aims:* There is limited data on health-related quality of life (HRQoL) in locally advanced rectal cancer. We assessed HRQoL before, during and after neoadjuvant chemoradiation, correlated this to corresponding clinician-reported adverse events (CR-AEs) and explored disparities between patients of Asian ethnicity versus Caucasians. Correlation between HRQoL and treatment response was also assessed. *Methods:* A consecutive sample of patients was recruited. HRQoL was assessed with the EORTC QLQ-C30 before chemoradiation, week three of chemoradiation and one-week pre-surgery. Clinical variables including CR-AEs were recorded at these time-points. Patients self-reported socio-demographic variables. Treatment response was assessed by the tumour regression grade. HRQoL data were analysed with multilevel models. *Results:* Fifty-one patients were recruited. HRQoL completion rates were ≥86%. Cognitive and role functioning worsened significantly during treatment. Emotional, role and social functioning improved significantly at pre-surgery. Fatigue and nausea/vomiting worsened during treatment while fatigue, appetite loss, diarrhoea and financial difficulties improved from treatment to pre-surgery. Almost 30% of the cohort were Asian ethnicity. Differences were found in multiple HRQoL domains between Asians and Caucasians, with Asians faring worse. Significant differences were evident in physical, role and cognitive functioning, and in seven out of the 8 symptom scales. The correlation between patient-reported outcomes and clinician-reported outcomes was weak, with diarrhoea having the strongest correlation (*r* = 0.58). Vomiting during treatment correlated with poor response, whilst baseline constipation correlated with good response. *Conclusion:* Chemoradiation for locally advanced rectal cancer affects multiple HRQoL domains. Our findings highlight the importance of psychological aspects of treatment. Significant differences were identified between the Asian and Caucasian populations, with Asians consistently performing worse. Poor correlations between patient and clinician reporting strongly support the inclusion of patient-reported outcomes in clinical studies. HRQoL domains of vomiting and constipation are potential biomarkers of treatment response.

## 1. Introduction

Colorectal cancer is the second most common cancer in females and third most common in males [1]. Rectal cancers comprise a third of colorectal cases. Of these, a third are locally advanced rectal cancer (LARC) which carry a poorer prognosis [2]. LARC is treated with trimodality therapy consisting of neoadjuvant radiation and chemotherapy, surgery and adjuvant chemotherapy [3]. Radiation is administered over 5–6 weeks with radiosensitising 5-fluorouracil or capecitabine. Following surgery, patients receive adjuvant chemotherapy. Despite trimodality therapy, recurrence rates are around 40%.

The quality of survivorship during and following trimodality treatment is an important issue. Although health-related quality of life (HRQoL) is central to treatment goals, there is limited data. The few studies on LARC, which utilize the EORTC QLQ-C30 and QLQ-CR38, are summarised below.

HRQoL was assessed before and after neoadjuvant chemoradiation, then 6- and 12-month post-surgery (*n* = 149) [4]. Physical, social functioning and body image decreased, while fatigue increased after treatment and normalised at 12 months. Global health status remained stable. There was a trend toward improvement in emotional functioning. Radiation-related toxicities of sexual dysfunction, faecal incontinence and impairment in bowel function were present at 12 months.

In another study (*n* = 42), 26 had neoadjuvant radiotherapy while 16 had post-operative radiotherapy [5]. HRQoL was assessed at the start and end of radiotherapy, and 4–6 weeks later. At the end of treatment, diarrhoea, fatigue and appetite loss significantly improved compared to pre-radiotherapy. There were small decreases in physical function and body image at the end of radiotherapy. All scores returned to baseline at 4–6 weeks.

Long-term HRQoL outcomes were assessed at 43 months post-neoadjuvant chemoradiation and radical surgery (*n* = 101), relative to the general population. Global health status, physical, role, social, emotional and cognitive functioning were not significantly different [6]. Social functioning was poorer; and constipation and diarrhoea were more prevalent. Interestingly, patients reported less pain, perhaps due to “response shift”, an adaptive psychological response [7]. 

Only one study (*n* = 50) assessed HRQoL outcomes during neoadjuvant chemoradiation, in a largely Caucasian population [8]. Nausea, vomiting, fatigue, dyspnoea, diarrhoea and urinary problems increased during treatment but returned to baseline levels post-treatment. Financial difficulties remained elevated. While completion rates for sexual function were poor, available data suggested it remained diminished post-treatment. Physical, role and global QoL decreased during chemoradiation and returned to baseline levels 4–6 weeks post-treatment. The authors noted that physician-rated toxicities may not capture patients’ experiences as fully as patient-reported outcomes.

Thus, neoadjuvant chemoradiation carries significant morbidity that may be under-recognised. Studies have focused on acute radiation-specific toxicities in a before-and-after design. Only one study investigated the impact on HRQoL during the treatment phase, when intervention may be possible. Potential under-reporting by physicians of toxicities may justify the inclusion of patient-reported HRQoL [9]. Further, physical toxicities are often the focus, while psychological impacts such as anxiety and depression are often neglected. There is also little literature on ethnic variability, and even less on South-East Asian, Middle-Eastern and South Asian populations. Available studies are in African–American and Japanese–American populations [10]. Given recognised cultural differences in perceptions of health care and symptom reporting, potential racial disparities may exist [11].

Tumour response to neoadjuvant therapy in LARC is measured by the tumour regression grade (TRG) scored on the surgical histopathological specimen. TRG has been shown to be prognostic for survival [12]. No studies have explored the correlation of HRQoL to TRG in LARC, although studies in metastatic colorectal cancer have correlated HRQoL to survival [13,14,15,16].

Our aims in this study were to address these deficiencies in the literature:

(1) Assess HRQoL before, during and after neoadjuvant chemoradiation.
(2) Assess the association between patient-reported HRQoL domains and corresponding clinician-reported adverse events (CR-AEs) and Eastern Cooperative Oncology Group (ECOG) performance status.
(3) To assess disparities between the Asian and Caucasian population.
(4) To assess whether HRQoL domain scores differ by tumour response as measured by the TRG.

## 2. Methods

Ethics approval was obtained from the Sydney South-West Area Health Service Ethics Committee (HREC/13/LPPL/158). Patients were prospectively recruited from Liverpool, Bankstown and Campbelltown Hospitals, Australia from 2014–2016. 

The inclusion criteria were: age > 18; tissue confirmation of rectal adenocarcinoma stage T3/T4 or node positive disease; no evidence of metastatic disease on computed tomography (CT) imaging of chest/abdomen/pelvis; treated with neoadjuvant chemoradiation. The treatment algorithm consisted of neoadjuvant chemotherapy with infusional 5-fluorouracil therapy or oral capecitabine concurrent with radiotherapy for 5–6 weeks, followed by surgery approximately 6–12 weeks post-treatment.

Data were collected within one week of commencing neoadjuvant chemoradiation (baseline), week three of treatment and post-treatment at one week before surgery. 

HRQoL was assessed with the EORTC QLQ-C30, a validated measure of core aspects of HRQoL and symptoms commonly experienced by cancer patients [17,18]. Standard scoring was used, yielding nine multi-item scales and six single-item scales [19]. For multi-item scales, the scale score was the average of the non-missing component items, as long as half or more items within the scale were completed; if more than half the items were missing, the scale was set to missing. Raw scores were linearly transformed to obtain scores ranging 0–100. On the functioning and global health status/QoL scales, a higher score indicated a better outcome, while on the symptom scales, a higher score indicated a worse outcome. 

Side effects of chemoradiation were recorded by physicians at these same time-points, graded using the Common Toxicity Criteria for Adverse Events (CTC-AE) version 4.0 [20], and included symptoms commonly experienced during chemoradiotherapy: diarrhoea, pain, fatigue, weight loss, nausea, vomiting, constipation, anorexia, insomnia and depression. 

ECOG performance status [21] and demographic data were also recorded. The Indian sub-continental population (South Asian), East Asian, South-east Asian, Middle-Eastern and Polynesian patients were classified as Asian in our analysis. The Australian, New Zealander, North American and European population were classified as Caucasian. 

TRG was scored by two specialist pathologists on the surgical specimens. Embedding the whole of the surgical tissue representing the original site of tumour was mandatory. All discrepancies between pathologists were resolved through consensus. The system used was that recommended by the American Joint Committee on Cancer (AJCC) using a four-point scale adapted from Ryan et al. with zero being complete tumour response with no viable tumour cells, and three being poor response with extensive residual tumour [2]. TRG 0–1 were classed as good responders and TRG 2–3 were classed as poor responders.

## 3. Statistical Methods

For each HRQoL outcome, multilevel models were used to compare HRQoL over the three time-points, and pairwise comparisons were then used to analyse differences between the time-points. Additionally, multilevel models using time-point, ethnicity and interaction between ethnicity and time-point as predictors were used to compare differences in outcomes between Asians and Caucasians at each time-point. This small study was descriptive and hypothesis-generating, and we used a *p*-value of 0.05 to highlight results of potential significance. We also used QLQ-C30 interpretation guidelines to assess clinical significance [22].

The association between patient-reported symptoms and corresponding clinician CTC-AE and ECOG performance status was assessed with Spearman’s correlation coefficient. Because in each case, patients and clinicians were assessing the same symptom, we considered correlations *r* ≥ 0.70 to be strong, *r* = 0.40–0.69 to be moderate, and *r* = 0.10–0.39 to be weak [23]. Boxplots were used to assess the range of patient-reported symptoms in each AE category.

Multilevel models using time-point, TRG and interaction between TRG and time-point as predictors were used to compare differences in domains of HRQoL between good and poor responders at each time-point.

## 4. Results

### 4.1. Study Population

Fifty-one patients were prospectively recruited. Completion rates for HRQoL questionnaires were 84% at baseline (43/51), 88% at week 3 (45/51) and 86% (38/44 still on study) pre-surgery. Reasons for coming off study included disease progression, withdrawal from study and patient refusal to undergo surgery. Lack of completion was generally due to an English language barrier and missed appointments. Toxicity data was recorded in 94% (48/51) of patients at baseline, 98% (50/51) at week three and 93% (41/44) pre-surgery. Table 1 summarises the patient characteristics.

### 4.2. Health-Related Quality of Life Domains and Symptom Scales of Study Population

Fatigue and nausea/vomiting increased significantly during treatment (*p* = 0.015 and *p* = 0.026 respectively). Symptoms of fatigue, appetite loss, diarrhoea and financial difficulties improved from treatment to pre-surgery (*p* = 0.001, *p* = 0.035, *p* = 0.0026 and *p* = 0.018 respectively). Cognitive and role function worsened significantly during treatment (*p* = 0.043 and *p* = 0.02 respectively). The latter improved significantly from treatment to pre-surgery (*p* = 0.03). Emotional functioning improved significantly from baseline to pre-surgery (*p* = 0.049). Social functioning worsened during treatment but improved significantly from both baseline and treatment levels to pre-surgery (*p* = 0.0078 and *p* = 0.019 respectively). All these changes were clinically important (Table S1) [22].

**Figure 1:**
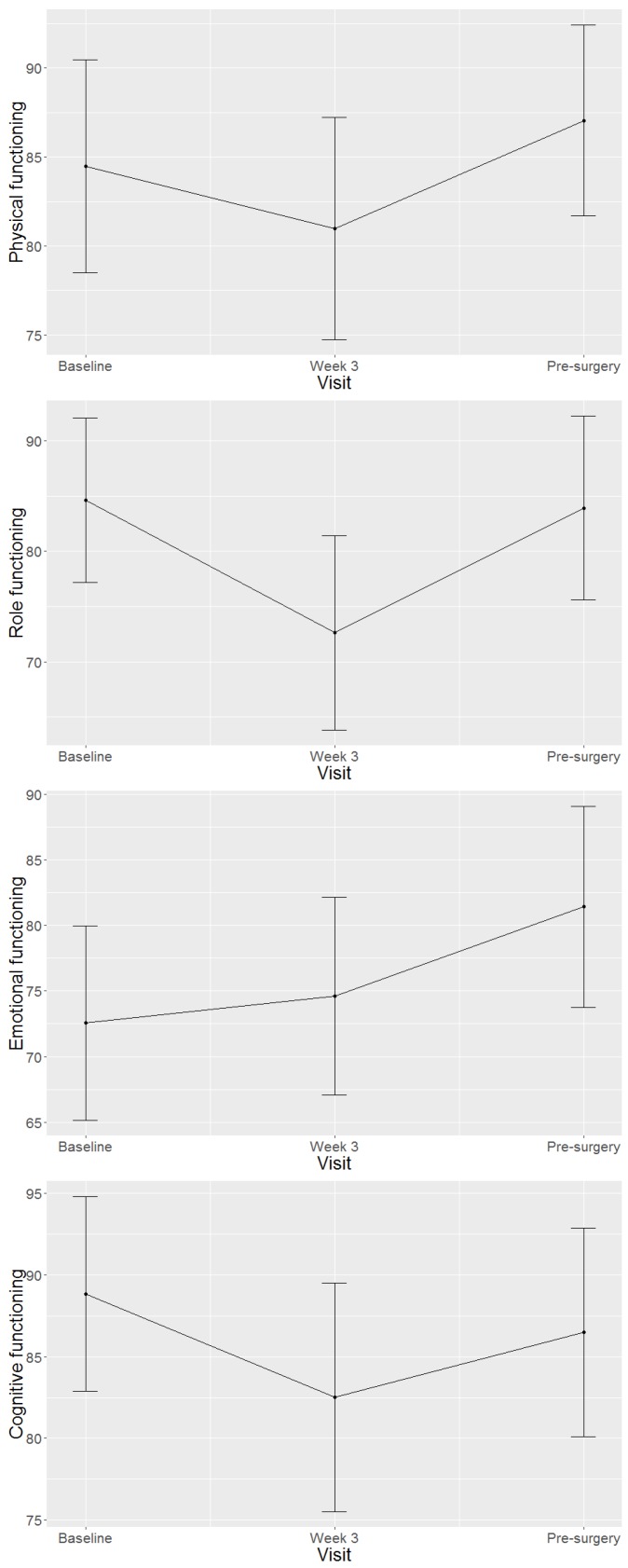

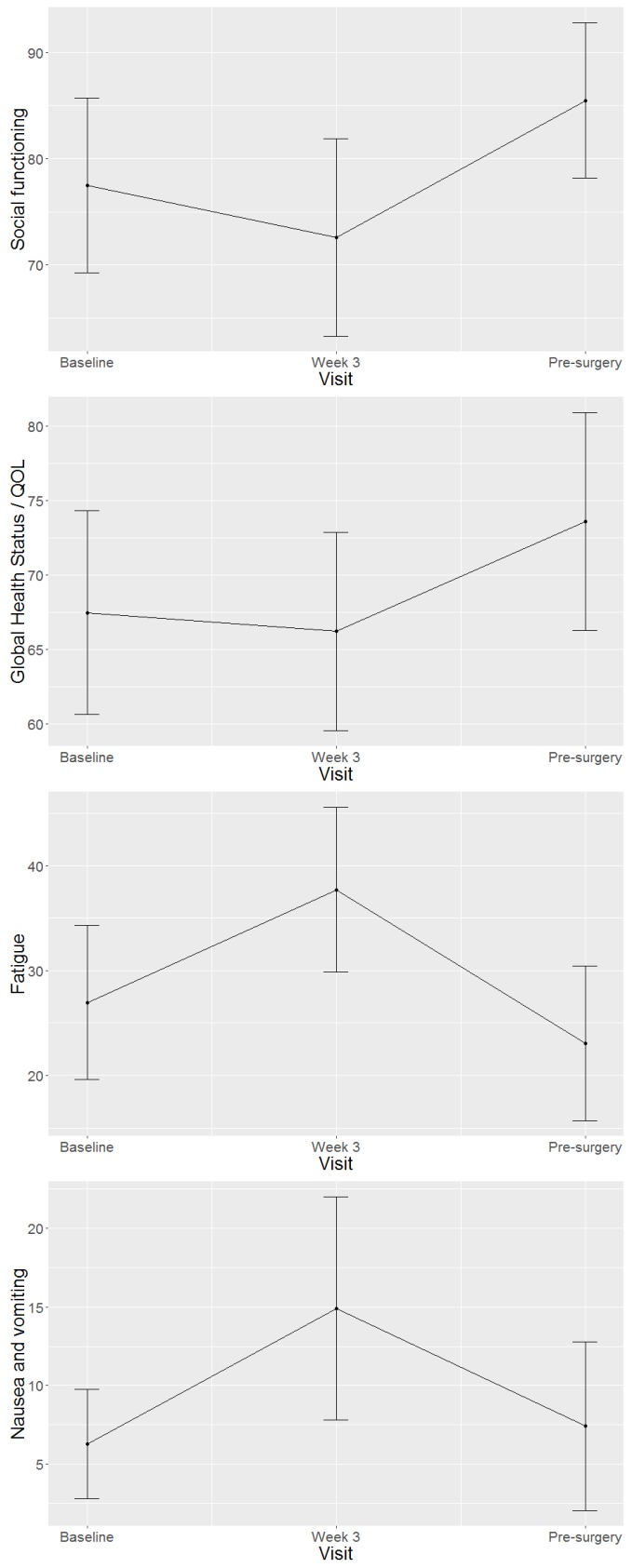

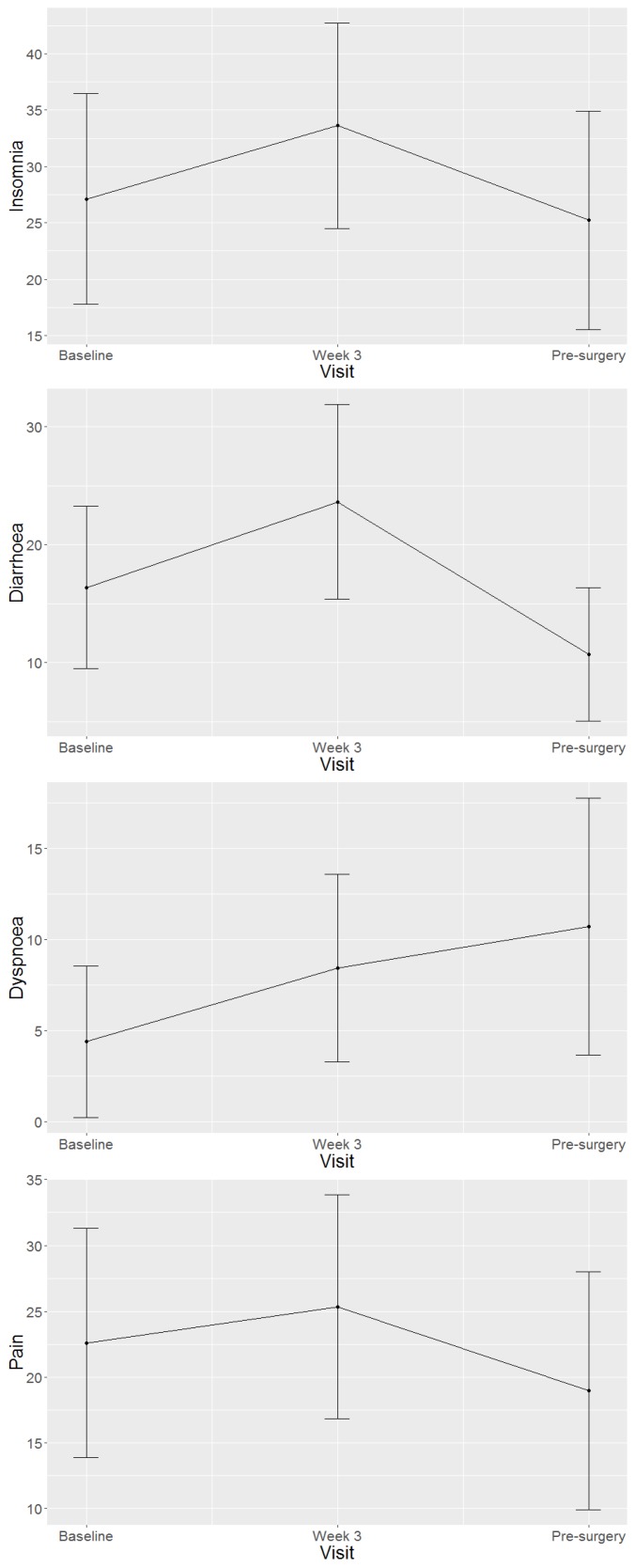

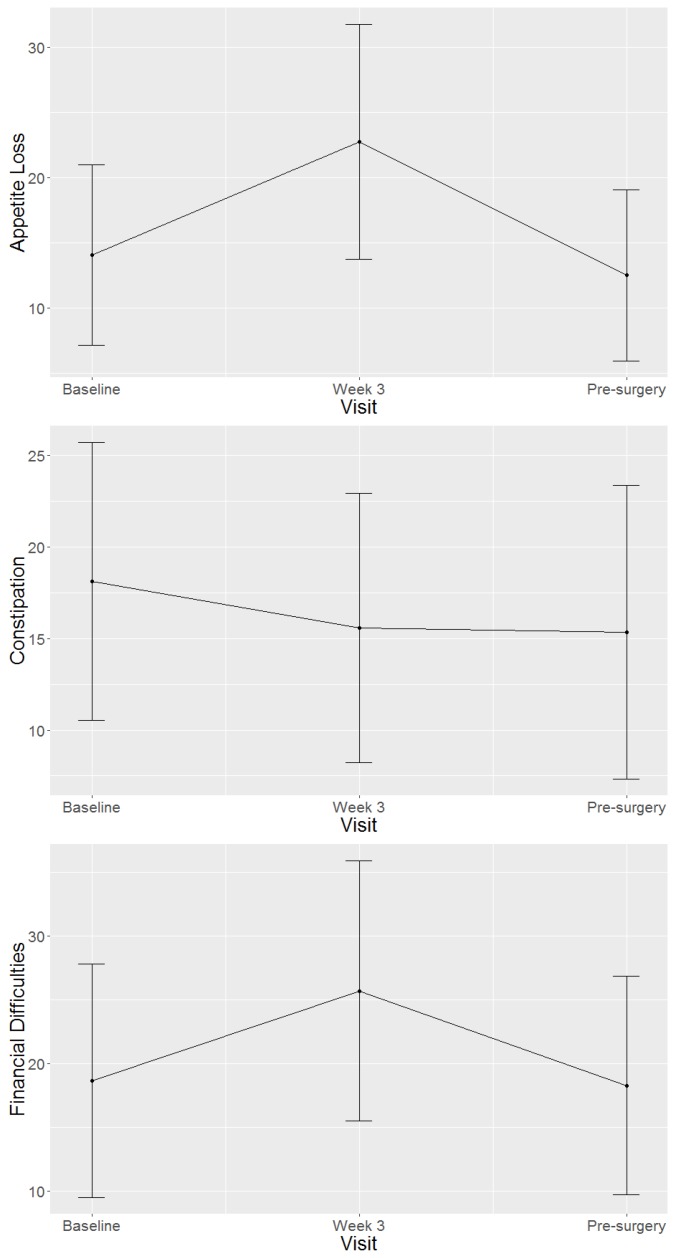
Line graphs illustrating the health-related quality of life scores at baseline, week three of treatment and pre-surgery for each domain. Means and confidence intervals are shown.

Figure 1 graphs the means and confidence intervals at the three time-points of each HRQoL domain. Table S1 in the Supplementary Material lists the means, confidence intervals, *p*-values and clinical significance of all pair-wise comparisons for all the HRQoL domains.

### 4.3. Health-Related Quality of Life Domains and Symptom Scales in Asian and Caucasian Populations

**Figure 2:**
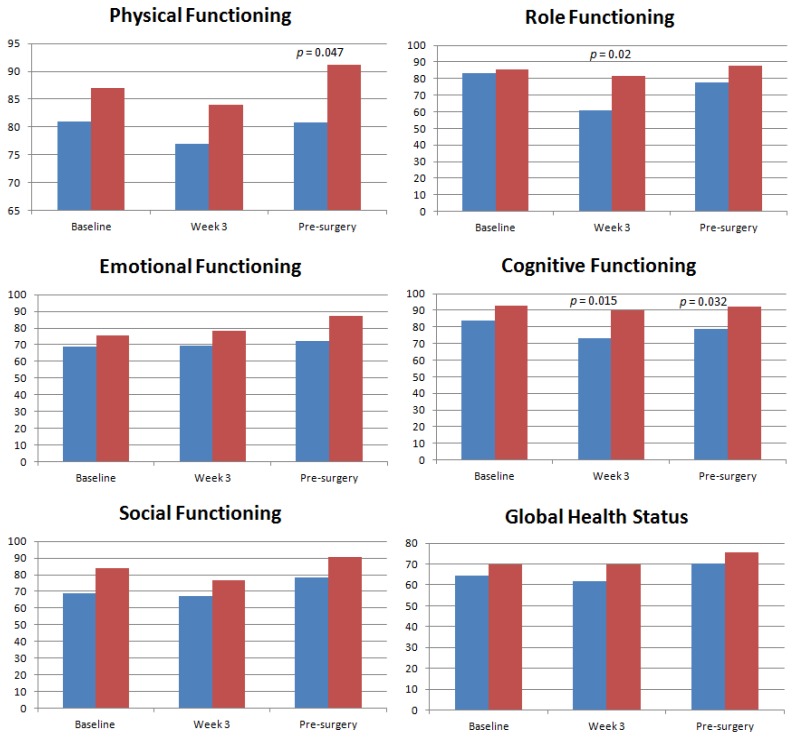

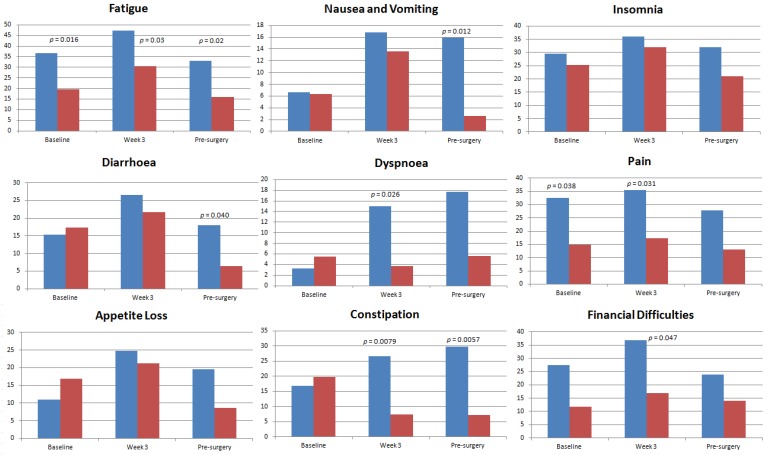
Health-related quality of life by ethnicity including functioning domains and symptom scales. Blue represents the Asian group; red represents the Caucasian group. The *p*-values are provided for significant differences between Asians and Caucasians at that time-point, based on pair-wise comparisons.

Differences were found in almost all the HRQoL domains between Asians and Caucasians, with Asians faring significantly worse. The bar graphs in Figure 2 show the mean scores between the two groups and the significant pair-wise comparisons (all of which were clinically significant). Means and confidence intervals for each domain and ethnic group are reported in Table S2 in the Supplementary Material, along with *p*-values and clinical significance of all pair-wise comparisons.

For functional scales, the Caucasian group on average had higher scores at baseline and a marked improvement pre-surgery while the Asian group had consistently lower scores and did not improve pre-surgery. Statistically and clinically significant pair-wise comparisons were evident in physical functioning at pre-surgery, role functioning at week three and in cognitive function at week three and pre-surgery.

Seven of the eight symptom scales showed statistically and clinically significant differences between Asians and Caucasians; in each case Asians reported higher levels of symptoms. Fatigue was worse in Asians at all three time-points. Pain was worse at baseline and during treatment in Asians. Dyspnoea was worse during treatment in Asians, and financial difficulties were greater. Constipation was also worse during treatment and at pre-surgery. Nausea and vomiting, as well as diarrhoea were worse in Asian patients at pre-surgery.

### 4.4. Adverse Events and Correlation between Patient-Reported Outcomes and Clinician-Reported Outcomes

Adverse events and ECOG performance status are shown in Table 2, most adverse events being grade 1. There were no grade 4 toxicities, nor ECOG level 4.

**Figure 3:**
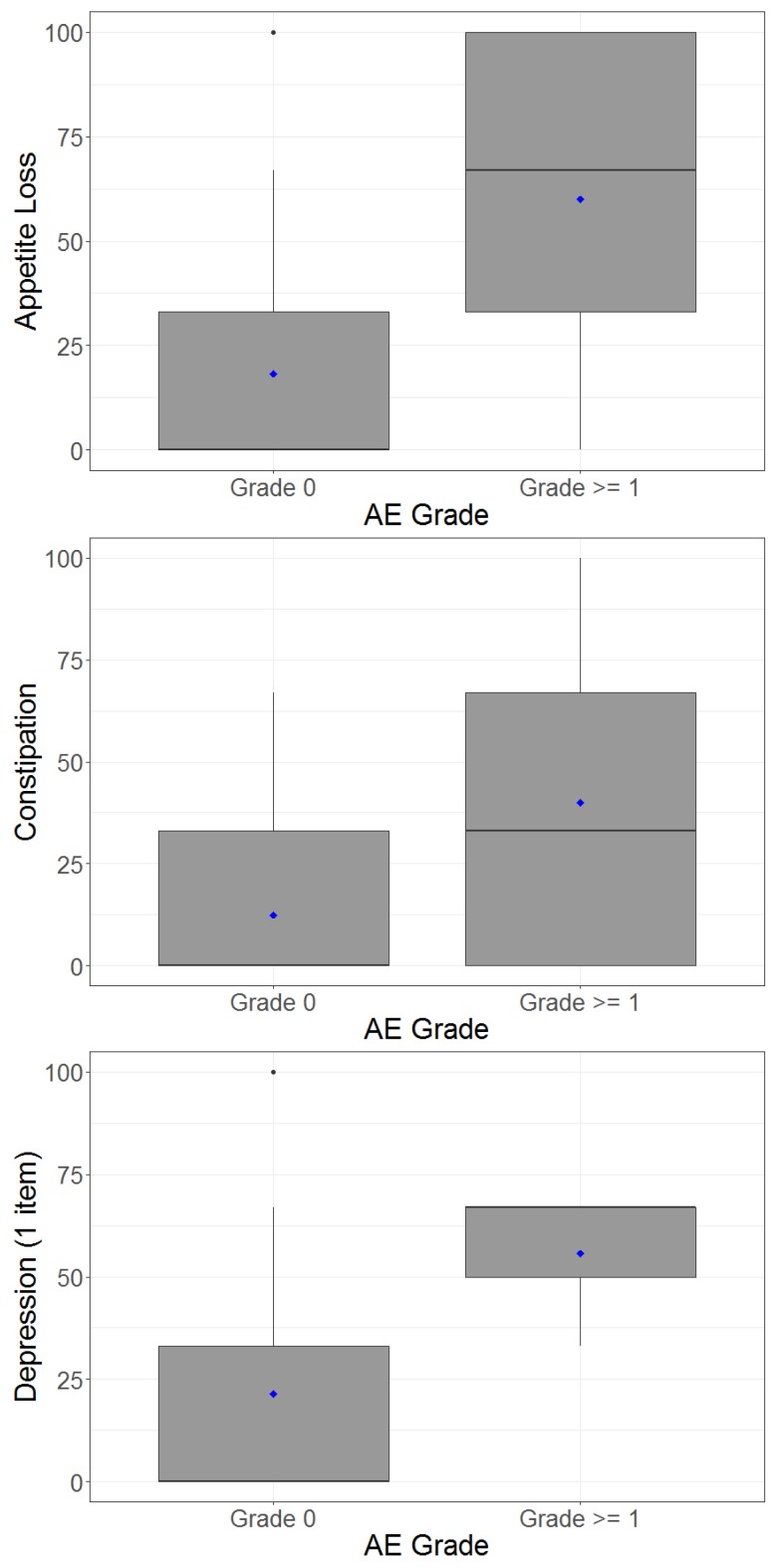

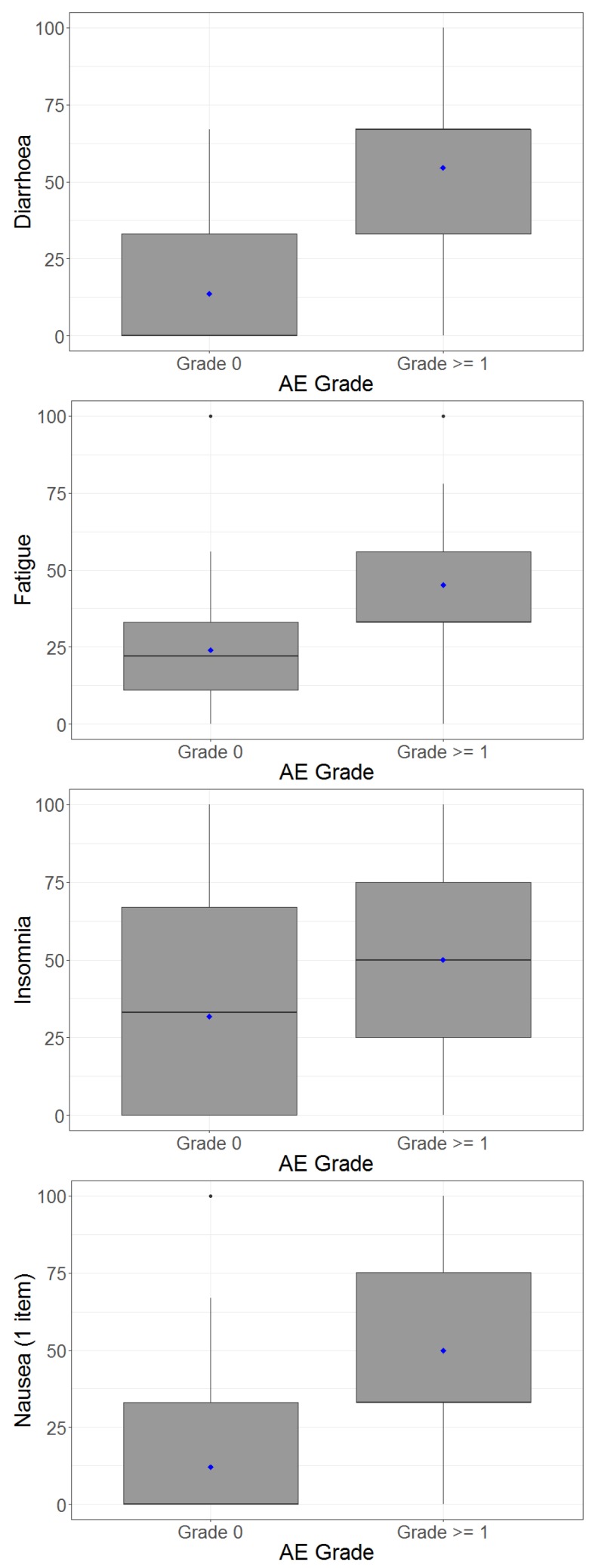

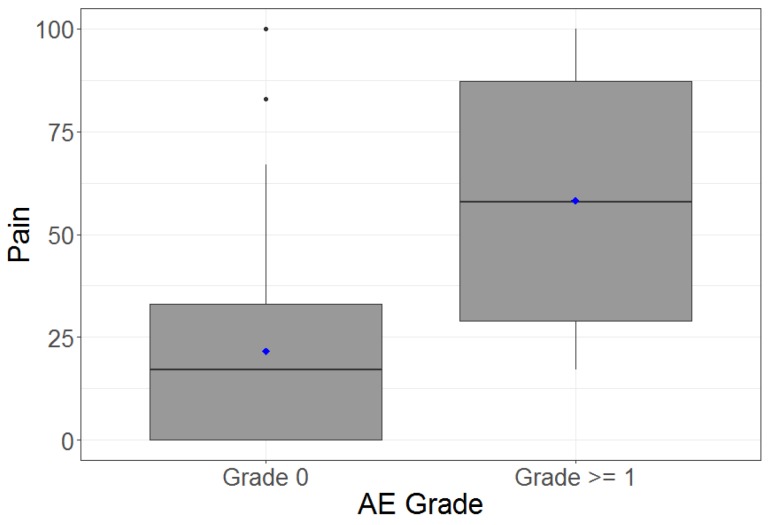
Box and whisker plots showing the patient-reported outcomes on the y-axis plotted against clinician-reported adverse events on the x-axis, grouped into grade 0 or grade ≥1. The boxes represent means (bold lines), upper and lower quartile scores while whiskers represent the maximum and minimum scores.

Diarrhoea and nausea displayed relatively strong correlations between patient-reported symptoms and CTC-AE, while fatigue, anorexia, pain and depression were moderately correlated. Insomnia had a very poor correlation (Table 3). Table 3 also shows mean patient-reported outcomes for patients rated by clinician as not having adverse events versus those having any grade of adverse event. Figure 3 illustrates the box and whisker plots of patient-reported outcomes plotted against clinician-reported adverse events. This highlights the extent of possible disagreement between patients and clinicians in reporting symptoms. For example, while diarrhoea had the strongest correlation (*r* = 0.58) and a clear difference in mean patient-reported scores between CTC-AE grade 0 versus grade 1 or more, some patients assigned grade 0 reported quite high levels of diarrhoea while some graded 1 or more reported no diarrhoea. These discrepancies were more common for all other domains, as these all had lower correlation.

### 4.5. Health-Related Quality of Life Domains and Tumour Response

TRG was available in 48 out of 51 patients. Three patients did not proceed to surgery. TRG breakdown was as follows: 17% TRG 0, 31% TRG 1, 44% TRG 2 and 8% TRG 3. This corresponds to 48% good responders and 52% poor responders. Poor responders had significantly worse vomiting on treatment compared to good responders (*p* = 0.032). Good responders had more constipation at baseline compared to poor responders (*p* = 0.046). The differences in means of the HRQoL of good and poor responders are shown in Table S3.

## 5. Discussion

We found chemoradiation for LARC affected multiple HRQoL domains. Cognitive and role functioning deteriorated with treatment, emotional functioning improved from baseline to pre-surgery, while role functioning improved from treatment to pre-surgery. Not much is known about cognitive function during neoadjuvant chemoradiation, although impairment in cognition is a well-recognised side effect of chemotherapy and long-term symptom in colorectal cancer survivors [24]. The trend in role function is consistent with findings by Herman et al. [8]. Pucciarelli et al. also shows a trend toward an improvement in emotional functioning, though this was at many months post-treatment [4]. This may be explained by an adjustment to cancer diagnosis, improvement in physical symptoms and psychological adaptation. 

Social functioning scores decreased during chemoradiation, but improved significantly from both baseline and treatment levels to pre-surgery. These findings are consistent with the literature, which has reported a fall in social functioning during or immediately post-treatment, then normalisation of these scores post-treatment [4,5,8]. However, these studies reported post-treatment scores either at 4–6 weeks, when radiation toxicities would have receded, or many months post-treatment when adjuvant chemotherapy is completed and stoma reversed. Timing of surgery was not specified. We have reported scores 6–12 weeks post-treatment, which is an important milestone in the treatment course before surgery.

We found that global health status remained unchanged. Herman et al. [8] reports a fall in global health scores but these normalised 4–6 weeks post-treatment. All remaining studies similarly reported no significant changes [4,5]. Consistent with the literature [4,5,8], our reported symptoms of fatigue, appetite loss and diarrhoea significantly increased during neoadjuvant treatment. 

There were marked differences identified at various time-points between the Asian and Caucasian populations, with Asians consistently faring worse with poorer function and more symptoms. The reasons for poorer HRQoL in Asians during and after treatment could be due to cultural differences in symptom reporting, perceptions of the health system, adherence to Western medicines, knowledge and compliance [10]. There are also reported inter-ethnic differences in metabolism, however no differences were found in drug pharmacokinetics between Caucasians and Japanese [11,25,26]. There is also no data to suggest that Asians have worse biology, though they may present later, reflecting socio-demographical differences [27]. However, we note that the disease-related symptoms of diarrhoea and constipation are not worse in our Asian cohort at presentation. 

Most previous studies have focused on effects of chemotherapy, while some included patients with rectal cancer undergoing multimodal therapy [28]. A core set of symptoms identified include nausea, fatigue, pain, depression and dyspnoea. We included these in our clinician assessment. Adverse events in our study were mostly grade 1. There was a 2% rate of grade 3 diarrhoea and no grade 4 toxicities. These clinician-reported toxicity data were consistent or slightly lower than reported [8]. 

We found a range of correlations between clinician-reported and patient-reported outcomes. Diarrhoea had the strongest correlation with a coefficient of 0.58. Even so, some patients assigned grade 0 reported high levels of diarrhoea while some graded ≥1 reported no diarrhoea. These discrepancies were more common in all other symptoms with poorer correlations. Insomnia has a particularly poor correlation (*r* = 0.06). These findings are consistent with the literature in other tumour types [29]. A retrospective subgroup study (*n* = 65) looking at patient and clinician-reported outcomes during chemoradiation in LARC reported on concordance between incidence of symptoms and found a correlation of 0.64 for diarrhoea [30]. 

Our findings strongly support the inclusion of patient-reported outcomes in clinical studies as under-recognition of patient symptoms by clinicians reduces the potential for intervention. Routine assessment of patient-reported outcomes can achieve clinical benefits when used to inform patient care, as shown in a randomised controlled study of outpatients undergoing chemotherapy [31,32]. 

To our knowledge, this is the first study that has attempted to correlate HRQoL and tumour response, which is prognostic of survival in LARC. Studies in metastatic colorectal cancer indicate that HRQoL are predictive of survival. We found that worse vomiting on treatment correlated to poor response. The presence of constipation at baseline predicted for a good response. We hypothesise that worse vomiting may result in decreased tolerability and absorption of systemic radiosensitising therapy, or may be related to the tumour cytokine milieu. Constipation at baseline may be a reflection of a responsive type of symptomatic tumour burden. 

While small sample size is a limitation, features of our study that add to the scant available evidence include reporting of serial measurements, selecting key clinically relevant time-points, comparison of clinician and patient-reported symptoms including psychological aspects of treatment. Importantly, we explored potential ethnic differences. We have also shown several HRQoL domains to correlate with tumour response, supporting increasing literature in the metastatic setting that demonstrate HRQoL to predict for survival. However, we acknowledge that not including the EORTC QLQ-CR38 will likely miss some important symptoms.

## 6. Conclusions

Our study provides much needed information about the symptom experience of patients with LARC, about which relatively little is known yet it is increasingly recognised as being key to patient-centred care. Poor correlations were found between patient and clinician reporting, which strongly support the inclusion of patient-reported outcomes in clinical studies. Importantly, we have shown marked ethnic disparities, which deserve further exploration in a larger study, and consideration of their causes and implications. Further exploration of HRQoL with tumour response with larger patient numbers, and correlating with long-term endpoints of survival, will be of clinical benefit.

## Figures and Tables

**Table 1:** Demographics and clinical profile of patient sample at baseline (*n* = 51).

Variable	*n*	Percent (%)
Age ˂65 ≥65	29 22	57 43
Gender Male Female	38 13	75 25
Ethnicity Caucasian Asian	36 15	71 29
Tumour stage T2 T3 T4	4 42 5	8 82 10
Nodal stage N0 N1 N2	7 20 24	14 39 47
Smoking status Yes No	21 30	41 59
BMI ˂30 ≥30	37 13	73 25
ECOG 0 1	36 15	71 29

**Table 2:** Adverse events as graded by Common Toxicity Criteria (CTC-AE) and Eastern Cooperative Oncology Group (ECOG) performance status at week three of chemoradiation, *n* = 50.

CTC-AE Specific Toxicity or ECOG *	Grade 0	Grade 1	Grade 2	Grade 3
Diarrhoea (%)	76	18	4	2
Pain (%)	90	8	2	0
Fatigue (%)	40	60	0	0
Weight loss (%)	94	6	0	0
Nausea (%)	76	22	2	0
Vomiting (%)	96	4	0	0
Constipation (%)	90	10	0	0
Anorexia (%)	88	10	2	0
Insomnia (%)	94	6	0	0
Depression (%)	92	8	0	0
ECOG performance status (%)	56	44	0	0

* no patients experienced grade 4 for any CTC-AE toxicity or were graded as ECOG = 4.

**Table 3:** Association between patient-reported outcomes and corresponding clinician-reported adverse events as graded by Common Toxicity Criteria (CTC-AE) at week three of chemoradiation.

Clinician-Rated Outcome	Patient-Reported Outcome (PRO)	Spearman’s Correlation	Mean (SD) PRO for Patients with CTC AE Toxicity Grade 0 ^†^	Mean (SD) PRO for Patients with CTC AE Toxicity Grade ≥ 1 ^†^
Diarrhoea	Diarrhoea	0.58	13.7 (18.6)	54.6 (30.8)
Nausea	Nausea	0.54	12.1 (23.3)	50.0 (36.2)
Fatigue	Fatigue	0.44	24.2 (25.5)	45.2 (24.9)
Anorexia	Appetite loss	0.35	18.3 (26.1)	60.0 (43.5)
Depression	Depression	0.32	21.4 (26.4)	55.6 (19.2)
Pain	Pain	0.31	21.5 (26.2)	58.3 (39.7)
Constipation	Constipation	0.24	12.5 (18.0)	40.0 (43.5)
Insomnia	Insomnia	0.06	31.8 (31.7)	50.0 (70.7)

**^†^** Patient-reported outcome item or multi-item scale that most closely corresponds to the clinician-reported outcome.

## Supplementary Materials

The following are available online at https://www.mdpi.com/2072-6694/11/9/1263/s1, Table S1: Health-related quality of life domains and symptom scales at baseline, week three of chemoradiation and pre-surgery, Table S2: Health-related quality of life domains and symptom scales at baseline, week three of chemoradiation and pre-surgery in Asian and Caucasian groups, Table S3: Health-related quality of life domains and symptom scales at baseline, week three of chemoradiation and pre-surgery in good and poor responders.
