# Supplementary material for: Health-Related Quality of Life during Chemoradiation in Locally Advanced Rectal Cancer: Impacts and Ethnic Disparities
Source: Cancers (Basel). 2019 Aug 28;11(9):1263. doi: 10.3390/cancers11091263 (PMC6770309; doi:10.3390/cancers11091263)
Supplement: Supplementary file 1 [file cancers-11-01263-s001.pdf]

SupplementaryMaterials

Health-Related Quality of Life during Chemoradiation in Locally Advanced Rectal Cancer: Impacts and Ethnic Disparities

Stephanie Hui-Su Lim, Emilia Ip, Weng Ng, Wei Chua, Ray Asghari, Aflah Roohullah, Joseph Descallar, Christopher Henderson, Kevin Spring, Paul de Souza and Madeleine T. King

Table S1. Health-related quality of life domains and symptom scales at baseline, week 3 of chemoradiation and pre-surgery. Data shown are estimates and their 95% confidence intervals based on multilevel models. The *p*-values of pair-wise comparisons based on multilevel models are shown, with clinical importance indicated. T1 = baseline, T2 = week 3 of treatment and T3 = presurgery.

HRQoL Domain	Baseline (*n* = 43)	Week 3 (*n* = 45)	Pre-Surgery (*n* = 38)	Pair Wise Comparison *p*-Values and Clinical Importance [1]		
				T1 & T2	T2 & T3	T1 & T3
**Functional Scales**						
Physical functioning	84.5 (78.5, 90.5)	81.0 (74.8, 87.2)	87.2 (81.7, 92.4)	0.34 trivial	0.07 small	0.15 small
Role functioning	84.6 (77.2, 92.1)	72.6 (63.8, 81.4)	83.9 (75.6, 92.2)	0.02 small	0.03 small	0.86 trivial
Emotional functioning	72.6 (65.2, 79.9)	74.6 (67.1, 82.2)	81.4 (73.8, 89.1)	0.63 trivial	0.11 small	0.049 small
Cognitive functioning	88.8 (82.9, 94.8)	82.5 (75.5, 89.5)	86.5 (80.1, 92.9)	0.043 small	0.16 small	0.22 trivial
Social functioning	75.5 (69.3, 85.7)	72.6 (63.3, 81.9)	85.5 (78.2, 92.8)	0.31 trivial	0.0078 medium	0.019 small
Global health status/QOL	67.5 (60.6, 74.3)	66.2 (59.6, 72.8)	73.6 (66.2, 80.9)	0.76 trivial	0.088 small	0.16 small
**Symptom Scales**						
Fatigue	26.9 (19.6, 34.3)	37.7 (29.8, 45.5)	23.1 (15.7, 30.4)	0.015 medium	0.001 medium	0.17 trivial
Nausea and vomiting	6.3 (2.8, 9.8)	14.9 (7.8, 22)	7.4 (2.1, 12.8)	0.026 small	0.079 small	0.63 trivial
Pain	22.6 (13.9, 31.3)	25.3 (16.8, 33.8)	19.0 (9.9, 28)	0.54 trivial	0.18 small	0.45 trivial
Dyspnoea	4.4 (0.3, 8.5)	8.4 (3.3, 13.6)	10.7 (3.7, 17.7)	0.20 trivial	0.42 trivial	0.056 small
Insomnia	27.1 (17.8, 36.5)	33.6 (24.5, 42.7)	25.2 (15.6, 34.9)	0.19 small	0.11 small	0.72 trivial
Appetite loss	14.1 (7.1, 21)	22.8 (13.7, 31.8)	12.5 (6, 19.1)	0.058 small	0.035 small	0.65 trivial
Constipation	18.1 (10.5, 25.7)	15.6 (8.2, 22.9)	15.4 (7.3, 23.4)	0.57 trivial	0.96 trivial	0.57 trivial
Diarrhoea	16.4 (9.5, 23.3)	23.6 (15.4, 31.9)	10.7 (5.1, 16.4)	0.14 small	0.0026 medium	0.18 small
Financial difficulties	18.7 (9.5, 27.8)	25.7 (15.5, 35.9)	18.3 (9.7, 26.9)	0.13	0.018	0.92

Interpretation guidelines provided by Cocks et al. (2012) [1] specify relative sizes of clinical important change: large, medium, small. Changes smaller than this are considered clinically trivial. Thresholds between categories may vary among HRQOL domains and for improvement/deterioration. Not available for Financial domain.

Table S2. Health-related quality of life domains and symptom scales at baseline, week 3 of chemoradiation and pre-surgery in Asian and Caucasian groups. Data shown are estimates and their 95% confidence intervals based on multilevel models. The *p*-values of pair-wise comparisons based on multilevel models are shown, with clinical importance indicated [1]. T1 = baseline, T2 = week 3 of treatment and T3 = presurgery.

HRQoL Domain	Baseline			Week 3			Pre-Surgery		
	**Asian (*n* = 19)**	**Caucasian (*n* = 23)**	***p*-Value**	**Asian (*n* = 20)**	**Caucasian (*n* = 25)**	***p*-Value**	**Asian (*n* = 14)**	**Caucasian (*n* = 24)**	***p*-Value**
**Functional Scales**									
Physical functioning	81.0<br>(71.9, 90)	87.0<br>(79.1, 94.9)	0.32<br>small	76.9<br>(67.6, 86.2)	84.0<br>(75.7, 92.2)	0.26<br>trivial	80.8<br>(72.8, 88.7)	91.2<br>(84.7, 97.7)	0.047<br>small
Role functioning	83.5<br>(72.3, 94.7)	85.4<br>(75.4, 95.3)	0.81<br>trivial	60.9<br>(48.4, 73.4)	81.6<br>(70.7, 92.4)	0.02<br>medium	77.7<br>(64.5, 90.9)	88.0<br>(77.5, 98.4)	0.23<br>small
Emotional functioning	68.8<br>(57.7, 79.8)	75.4<br>(65.5, 85.2)	0.37	69.5<br>(58.3, 80.7)	78.5<br>(68.5, 88.4)	0.24	72.3<br>(60.8, 83.9)	87.2<br>(77.8, 96.7)	0.051
Cognitive functioning	83.7<br>(74.9, 92.4)	92.7<br>(85.0, 100)	0.13<br>medium	73.0<br>(63.1, 83)	89.7<br>(81, 98.5)	0.015<br>large	78.5<br>(69.1, 88.0)	92.1<br>(84.2, 100)	0.032<br>medium
Social functioning	69.0<br>(57.1, 80.9)	84.1<br>(73.5, 94.8)	0.063<br>medium	67.0<br>(53.2, 80.8)	76.8<br>(64.6, 89.1)	0.29<br>small	78.6<br>(67.2, 89.9)	90.5<br>(81.4, 99.6)	0.11<br>medium
Global health status/QOL	64.5<br>(54.4, 74.6)	69.7<br>(60.6, 78.8)	0.45<br>small	61.7<br>(51.8, 71.6)	69.8<br>(61, 78.6)	0.23<br>small	70.1<br>(58.2, 82.1)	75.8<br>(66.7, 85)	0.45<br>small
**Symptom Scales**									
Fatigue	36.9<br>(26.5, 47.3)	19.6<br>(10.4, 28.8)	0.016<br>large	47.2<br>(35.9, 58.4)	30.4<br>(20.4, 40.4)	0.030<br>medium	33.1<br>(22.1, 44.2)	16.0<br>(7.1, 25)	0.020<br>medium
Nausea and vomiting	6.6<br>(1.3, 11.9)	6.3<br>(1.6, 10.9)	0.92<br>trivial	16.8<br>(6.2, 27.4)	13.6<br>(4.1, 23.1)	0.66<br>trivial	15.9<br>(8, 23.9)	2.6<br>(0, 9)	0.012<br>medium
Pain	32.6<br>(20.1, 45.1)	14.8<br>(3.6, 26)	0.038<br>medium	35.5<br>(23.2, 47.7)	17.4<br>(6.6, 28.3)	0.031<br>medium	27.8<br>(13.7, 41.8)	13.0<br>(2, 24)	0.10<br>medium
Dyspnoea	3.2<br>(0, 9.3)	5.5<br>(0, 11)	0.58<br>trivial	15.0<br>(7.5, 22.5)	3.7<br>(0, 10.2)	0.026<br>medium	17.7<br>(7.1, 28.2)	5.6<br>(0, 14.1)	0.078<br>medium
Insomnia	29.6<br>(15.7, 43.5)	25.3<br>(12.9, 37.8)	0.65<br>small	36.0<br>(22.3, 49.6)	32.0<br>(19.9, 44)	0.66<br>small	32.0<br>(16.4, 47.6)	21.1<br>(8.8, 33.3)	0.27<br>small
Appetite loss	11.0<br>(0.7, 21.4)	16.9<br>(7.7, 26)	0.40<br>small	24.7<br>(11.2, 38.2)	21.2<br>(9.2, 33.2)	0.70<br>trivial	19.6<br>(9.4, 29.7)	8.7<br>(0.6, 16.8)	0.099<br>small
Constipation	16.8<br>(6.1, 27.5)	19.6<br>(9.9, 29.2)	0.70<br>trivial	26.4<br>(15.9, 36.9)	7.3<br>(0, 16.6)	0.0079<br>large	29.8<br>(17.1, 42.4)	7.3<br>(0, 16.8)	0.0057<br>large
Diarrhoea	15.3<br>(5.1, 25.5)	17.3<br>(8, 26.6)	0.77<br>trivial	26.5<br>(14.1, 38.9)	21.7<br>(10.7, 32.7)	0.56<br>small	18.0<br>(9.3, 26.7)	6.4<br>(0, 13.1)	0.040<br>medium
Financial difficulties	27.5<br>(14.2, 40.8)	11.8<br>(0, 23.7)	0.083	36.9<br>(22, 51.7)	17<br>(4.1, 29.8)	0.047	23.9<br>(10.4, 37.3)	13.9<br>(2.8, 25)	0.26

Interpretation guidelines provided by Cocks et al. (2011) [1] specify relative sizes of clinical important differences: large, medium, small. Differences smaller than this are considered clinically trivial. Thresholds between categories may vary among HRQOL domains and for improvement/deterioration. Not available for Emotional and Financial domain.

Table S3. Health-related quality of life domains and symptom scales at baseline, week 3 of chemoradiation and pre-surgery in good and poor responders. Data shown are estimates, standard error and their 95% confidence intervals based on multilevel models. The *p*-values of pair-wise comparisons based on multilevel models are shown. Time 1 = baseline, Time 2 = week 3 of treatment and Time 3 = presurgery.

HRQoL Domain	Good Responder (TRG 0-1)		Poor Responder (TRG 2-3)		Difference			
	Mean	Standard Error	Mean	Standard Error	Mean	Lower 95% CI	Upper 95% CI	*p*-value
**Functional Scales**								
**Physical Function**								
Time 1	85.19	21.15	83.03	21.94	3.68	−9.01	16.36	0.5621
Time 2	84.67	16.34	79.39	25.02	5.88	−7.26	19.02	0.3712
Time 3	87.00	17.50	92.50	16.12	−5.96	−17.34	5.42	0.296
**Role Function**								
Time 1	81.30	6.29	89.22	5.74	−7.92	−24.80	8.96	0.3543
Time 2	82.44	6.01	68.72	5.74	13.72	−2.75	30.19	0.1016
Time 3	82.03	6.00	88.38	6.42	−6.35	−23.76	11.06	0.4713
**Emotional Function**								
Time 1	74.36	5.68	70.13	5.22	4.23	−11.09	19.54	0.585
Time 2	81.36	5.47	69.84	5.22	11.53	−3.49	26.55	0.1308
Time 3	82.30	5.67	81.32	5.72	0.98	−15.00	16.96	0.9035
**Cognitive Functioning**								
Time 1	85.42	4.90	91.15	4.57	−5.73	−19.08	7.62	0.395
Time 2	85.53	4.78	79.18	4.57	6.35	−6.83	19.53	0.3401
Time 3	85.50	4.89	86.58	4.86	−1.08	−14.81	12.64	0.8756
**Social Functioning**								
Time 1	73.00	6.20	80.63	5.71	−7.63	−24.39	9.13	0.368
Time 2	76.62	5.99	71.17	5.71	5.45	−11.01	21.92	0.5117
Time 3	83.23	6.19	87.21	6.22	−3.97	−21.40	13.46	0.652
**Global health status/QOL**								
Time 1	64.90	5.18	69.63	4.72	−4.72	−18.62	9.17	0.5017
Time 2	73.44	4.95	61.37	4.72	12.08	−1.49	25.64	0.0803
Time 3	72.15	5.17	76.46	5.28	−4.30	−18.95	10.35	0.5616
**Symptom Scales**								
**Fatigue**								
Time 1	29.69	5.87	23.86	5.41	5.83	−10.04	21.70	0.4673
Time 2	35.65	5.66	38.78	5.41	−3.14	−18.71	12.44	0.6899
Time 3	25.13	5.86	21.51	5.90	3.62	−12.90	20.13	0.6646
**Nausea and Vomiting**								
Time 1	3.75	2.57	8.17	2.38	−4.43	−11.48	2.63	0.2129
Time 2	5.13	4.93	20.29	4.71	−15.16	−28.92	−1.39	0.0317
Time 3	7.89	3.76	7.66	3.94	0.23	−10.77	11.23	0.9668
**Pain**								
Time 1	27.26	6.76	20.05	6.23	7.21	−11.07	25.49	0.435
Time 2	21.67	6.52	28.67	6.23	−7.00	−24.95	10.94	0.4397
Time 3	26.44	6.75	15.04	6.79	11.40	−7.62	30.41	0.237
**Dyspnoea**								
Time 1	1.41	2.98	5.99	2.74	−4.57	−12.73	3.59	0.2646
Time 2	4.42	3.75	11.90	3.64	−7.47	−18.00	3.05	0.1597
Time 3	7.71	4.75	12.13	4.92	−4.42	−18.30	9.46	0.5224
**Insomnia**								
Time 1	31.86	6.99	22.58	6.45	9.28	−9.60	28.16	0.3317
Time 2	26.26	6.72	37.26	6.45	−11.00	−29.51	7.50	0.2405
Time 3	30.78	6.71	18.49	7.09	12.29	−7.08	31.65	0.211
**Appetite Loss**								
Time 1	11.66	5.54	14.10	5.08	−2.45	−17.36	12.46	0.7455
Time 2	12.85	5.31	27.11	5.08	−14.25	−28.84	0.33	0.0553
Time 3	13.99	5.30	11.40	5.63	2.60	−12.74	17.93	0.7377
**Constipation**								
Time 1	26.18	5.77	10.41	5.26	15.77	0.30	31.24	0.0458

Time 2	14.36	5.50	18.85	5.26	−4.49	−19.57	10.59	0.5567
Time 3	18.34	5.50	9.78	5.90	8.56	−7.42	24.54	0.2908
**Diarrhoea**								
Time 1	19.95	5.33	14.50	4.85	5.46	−8.82	19.74	0.4505
Time 2	18.16	5.08	27.21	4.85	−9.05	−22.96	4.87	0.2003
Time 3	13.30	5.33	7.50	5.45	5.80	−9.30	20.91	0.448
**Financial difficulties**								
Time 1	20.16	6.97	15.67	6.49	4.49	−14.50	23.48	0.6386
Time 2	25.20	6.79	21.20	6.49	4.00	−14.74	22.74	0.6717
Time 3	20.85	6.96	15.24	6.91	5.61	−13.91	25.13	0.5687

Abbreviation: confidence interval (CI), tumour regression grade (TRG).

Reference

1. Cocks, K., King, M.T.; Velikova, G.; Fayers, P.M.; Brown, J.M. Quality, interpretation and presentation of European Organisation for Research and Treatment of Cancer quality of life questionnaire core 30 data in randomised controlled trials. *Eur. J. Cancer* **2008**, *44*, 1793–1798.

© 2019 by the authors. Licensee MDPI, Basel, Switzerland. This article is an open access article distributed under the terms and conditions of the Creative Commons Attribution (CC BY) license (http://creativecommons.org/licenses/by/4.0/).
